# Microfluidic Cell Transport with Piezoelectric Micro Diaphragm Pumps

**DOI:** 10.3390/mi12121459

**Published:** 2021-11-27

**Authors:** Agnes Bußmann, Thomas Thalhofer, Sophie Hoffmann, Leopold Daum, Nivedha Surendran, Oliver Hayden, Jürgen Hubbuch, Martin Richter

**Affiliations:** 1 Fraunhofer EMFT Research Institution for Microsystems and Solid State Technologies, Hansastrasse 27d, 80686 Munich, Germany; thomas.thalhofer@emft.fraunhofer.de (T.T.); sophie.hoffmann@unibw.de (S.H.); nivedha.surendran@emft.fraunhofer.de (N.S.); martin.richter@emft.fraunhofer.de (M.R.); 2 MAB-Biomolecular Separation Engineering, Karlsruhe Institute of Technology, Fritz-Haber-Weg 2, 76131 Karlsruhe, Germany; juergen.hubbuch@kit.edu; 3 TranslaTUM—Central Institute for Translational Cancer Research, Technical University of Munich, Einsteinstrasse 25, 81675 Munich, Germany; leo.daum@tum.de (L.D.); oliver.hayden@tum.de (O.H.)

**Keywords:** micro diaphragm pump, microfluidic, micro dosing, cell transport, automated cell culture, passive spring valves

## Abstract

The automated transport of cells can enable far-reaching cell culture research. However, to date, such automated transport has been achieved with large pump systems that often come with long fluidic connections and a large power consumption. Improvement is possible with space- and energy-efficient piezoelectric micro diaphragm pumps, though a precondition for a successful use is to enable transport with little to no mechanical stress on the cell suspension. This study evaluates the impact of the microfluidic transport of cells with the piezoelectric micro diaphragm pump developed by our group. It includes the investigation of different actuation signals. Therewith, we aim to achieve optimal fluidic performance while maximizing the cell viability. The investigation of fluidic properties proves a similar performance with a hybrid actuation signal that is a rectangular waveform with sinusoidal flanks, compared to the fluidically optimal rectangular actuation. The comparison of the cell transport with three actuation signals, sinusoidal, rectangular, and hybrid actuation shows that the hybrid actuation causes less damage than the rectangular actuation. With a 5% reduction of the cell viability it causes similar strain to the transport with sinusoidal actuation. Piezoelectric micro diaphragm pumps with the fluidically efficient hybrid signal actuation are therefore an interesting option for integrable microfluidic workflows.

## 1. Introduction

The detailed experimental analysis of biological systems on a microscale is a common subject of current research. Within the last 30 years, micro fluidic systems developed rapidly and already offer solutions for various experimental setups [1,2]. Today, analysis on aggressively scaled devices is possible. However, the sample transport either relies on passive capillary forces or requires systems with bulky external pumps for active transport that often have a high power consumption and are connected with long tubing, which leads to a high dead volume [3,4,5,6]. The active transport in space-restricted situations, such as in clinical environments, is even more challenging. The integration of micropumps can offer a cost as well as an energy efficient on-chip solution for active sample transport.

Micropumps are not only of interest for the active transport in microfluidic setups, but can also offer a possibility to improve three-dimensional (3D) bioprinting. This technique enables the generation of complex structures that are of interest in pharmaceutical research, regenerative medicine, or the food industry [7,8]. For instance, the advantages of 3D-cultures compared to 2D-cultures for pharmaceutical research are demonstrated in several studies [9,10,11] and an industrial use of 3D cultures in pharmaceutical research is expected [7].

Bioprinting is widely researched as treatment of large skin injuries. In particular, large skin lesions can lead to poor healing, infection, or hypertrophic scars [12,13]. The state-of-the-art treatment is skin transplant from healthy areas. Even though meshing can increase the area of a graft tremendously [14], it is often problematic to harvest sufficient skin. Bioprinting of skin tissue is proposed as possible solution [15] including two main approaches: the generations of grafts in vitro that are cultivated and implanted into the wound and the in situ printing directly into the wound [16].

Fluid transport for the printing process can be more challenging for in situ printing compared to laboratory configurations. Similar to analysis systems, laboratory printing setups often rely on large pump systems. However, a printer used in a hospital environment is easier to imagine as a handheld device with small weight and ergonomic geometry. Hakimi et al. [17] present an in situ handheld skin printer. The device weighs less than 800 g and is able to print different cell materials, which allows for consecutive deposition of dermal and epidermal layers. The printability on rough wound surfaces is already shown in vivo.

Micro diaphragm pumps are a possible solution for active cell transport in the presented applications. There is a large amount of research conducted on micropumps and several actuation mechanisms have emerged [18,19,20]. A common actuation principle is piezoelectric actuation. It is popular due to its energy-efficiency, high attained forces, large range of applicable frequencies, and precise control [21]. Another common pump type is the electromagnetically actuated pump, which also allows for energy efficient actuation [22].

Energy-efficiency and the possibility for low-cost production as well as small size [20] make micropumps an ideal microfluidic actuator for cell transport, even in disposable application. However, currently there is little information on the interaction of micropumps with the dosed medium. In particular, cell solution is not usually transported and analysed. The only work known to the authors that discusses cell transport with a micro diaphragm pump describes the viability of cells after passage through an electromagnetic pump with diffuser nozzle valves [23]. Yamahata et al. [23] show their pump to be well adapted for cell transport, since the viability of both the tested Jurkat cells as well as the more delicate 5D10 hybridoma cells remains high after pumping. However, the fluidic performance of the pump is lower than observed for other micropumps: the 33 × 22 mm^2^ pump can transport 400 µL/min and shows a blocking pressure of 12 mbar [24]. The low backpressure capability is most likely partly caused by the diffuser nozzle valves. These valves additionally limit bubble tolerance, though the pump is able to transport bubbles without backpressure due to its compression ratio over 0.2 [24].

Even though a diffuser nozzle valve design is most likely less destructive for cells, it limits the fluidic performance of a pump system. The backpressure capability, bubble tolerance, and ability to transport fluids with a high viscosity such as bioinks can be improved with passive valves, e.g., flap or spring valves. However, the impact of such valves that constitute moving parts with sharp edges and a risk of squeezing the cells between the valve and valve seat is unknown to date. To enable cell transport for in situ bioprinting or on chip analysis application with high performance micropumps, we investigated the influence of our piezoelectric micro diaphragm pumps with spring valves on viable cell suspensions. The aim of the presented work is to examine the feasibility of cell transport with a micropump including passive valves to enable future developments of microfluidic devices with integrated automated fluid transport.

## 2. Materials and Methods

The pumps used for cell transport in this work are Fraunhofer EMFT’s stainless steel pumps. A fluidic characterisation of the samples is conducted before and after the cell transport experiments.

### 2.1. Piezoelectric Micro Diaphragm Pumps

A detailed evaluation of the pump type used in this work is given by Bußmann, Durasiewicz et al. [25]. The devices have a diameter of 20 mm and a height of 1.5 mm. The pumps consist of a stainless-steel pump body and a glued on piezoelectric disc actuator (PIC 151, diameter of 16 mm; thickness of 200 µm). The pump body includes two passive spring valves and the actuator diaphragm. All components are laser welded to the base plate. The pump chamber has a diameter of 18 mm and a height of approximately 100 µm to 150 µm, depending on the mechanical stress generated during the welding process and the electric tension applied during the mounting process of the piezoceramic actuator. The valves of the pump are 5 mm apart.

**Figure 1:**
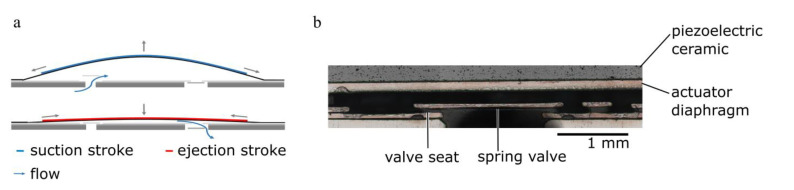
Piezoelectric micro diaphragm pump. (**a**) Functional principle: A negative filed moves the bending actuator upwards and sucks liquid through the passive inlet valve into the pump chamber. A positive electric field moves it towards the chamber bottom and pushes the fluid through the outlet valve. Figure adapted from Bußmann et al. [25]. (**b**) Section of the pump’s inlet valve including the spring valve and the valve seat that form the valve gap that is approximately 300 µm long and in the open state of the valve 50 µm high.

Fluid transport bases on the indirect piezoelectric effect (Figure 1). The exposure of the piezoelectric ceramic to an alternating high voltage signal leads to an oscillatory diaphragm movement. The resulting periodic expansion and reduction of the chamber volume in combination with the flow restriction of the passive spring valves leads to a directed fluid transport. The passive flap valves limit backflow and enable a high backpressure capability as well as high bubble tolerance. Therefore, they are indispensable for the fluidic performance of the micropump.

#### 2.1.1. Actuator Stroke Measurements

The actuator stroke is an important metric for micro membrane pumps. It is measured optically using a white light profilometer (Fries Research and Technology, Bergisch Gladbach, Germany, sensor range: 3 mm, sensitivity: 30 nm). A voltage sweep is executed ranging from 0.4 kV/mm to 2 kV/mm (amplifier SVR 500−3, piezosystem jena GmbH, Jena, Germany) and the respecting actor positions are detected. The sweep is repeated multiple times. Repetitive tests of the same pump show a measurement accuracy of 2 µm.

#### 2.1.2. Fluidic Characterisation

The pumps are characterized with both air as well as deionised (DI) water at room temperature. The actuation signal for these measurements is a sinusoidal alternating voltage from −0.4 kV/mm to 1.5 kV/mm, which equals −80 V to 300 V for the 200 µm thick actuator. Water flow is measured with Coriflow sensors (Bronkhorst, Kamen, Germany ML120V00: range 0.8 μL/min to 500 μL/min, accuracy: ±0.2% and Bronkhorst MINI CORI-FLOW M14: range 0.5 mL/min to 167 mL/min, accuracy: ±0.2%). The backpressure for both water and air characterisation is set with a pressure controller (Mensor, San Marcos, TX, USA CPC3000: range −50 kPa to 200 kPa, accuracy: ±0.05 kPa). During water measurements, the differential pressure over the pumps is measured with two piezoresistive pressure sensors (TDK Electronics, Munich, Germany, EPCOS Gauge pressure transducers AKR 1.000 C40: range 0.0 kPa–10o kPa, accuracy: ±0.6 kPa).

When transporting liquid media, it is always possible that small bubbles occur and the pump needs to be able to transport them through the chamber. We therefore test the pump’s backpressure capability with air at actuation frequencies for liquid transport (30 and 60 Hz). The pressure is increased by 2.5 kPa until the flow reaches zero.

Water characterisation of the pumps includes the frequency dependant flow at a constant pressure of 14 kPa in the range from 1 Hz to 50 Hz, the pressure dependant flow in the range from 0 kPa to 50 kPa, and pressure dependant leakage in the range from 5 kPa to 50 kPa.

#### 2.1.3. Single Stroke Characterisation

**Figure 2:**
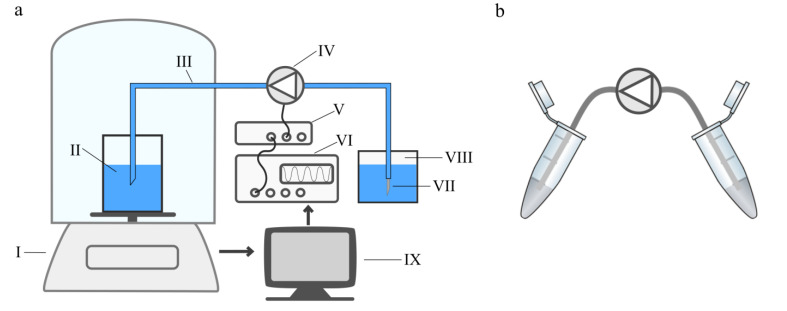
(**a**) Experimental setup of the gravimetric measurement to determine the single stroke volume for different actuation signals including a balance (I), the inlet reservoir (II), silicone tubing (III), the micro diaphragm pump (IV) driven with a piezo amplifier (V) and signal generator (VI), an outlet capillary (VII), and pressure equalized reservoir (VIII), as well as automated control and data acquisition (IX). (**b**) Setup of the cell transport experiments with an inlet- and outlet reservoir as well as the pump connected with silicone tubing. A picture of the two experimental setups is available in Figure A1 in Appendix A.

We evaluate the pump’s single stroke volume with DI water in a gravimetric measurement depicted in Figure 2. It is based on a setup introduced by Thalhofer et al. [26]. The inlet reservoir is placed on a precision scale (Sartorius, Göttingen, Germany, MC410S, resolution 100 µg) with the inlet tube immersed free-hanging in the liquid at all times. The pump, which is placed next to the balance, transports liquid from the inlet reservoir to the outlet. The actuation signal is triggered using an automated protocol. The single stroke signal is amplified with the SVR 500−3 (piezosystem jena GmbH, Jena, Germany). The fluid reservoirs are covered with Nitto SWT 10+R to minimize drift caused by condensation or evaporation. 

The weight of the outlet reservoir is detected before and after the dosage of one single stroke in order to calculate the dosed single stroke volume from the weight difference. The sampling frequency is 1 Hz and does not affect the measurement, since we detect the total weight of a single stroke and do not depict the time dependant weight change during the stroke. To account for the drift of the balance, we perform five scale measurements before a single pump stroke is performed with defined waveform and frequency. After an eight seconds break, another five post-trigger scale measurements are recorded. The weight difference before and after the pump stroke is evaluated and corrected for the average drift that is calculated based on the five consecutive measurements before and after the trigger.

### 2.2. Cell Transport

Experiments with cells are conducted with two different cell lines cultivated in suspension culture: K-562 human chronic myeloid leukaemia cells [27] (DSMZ GmbH, Braunschweig, Germany; DSMZ no.: ACC 10) of approximately 17 µm diameter and Jurkat T cell leukaemia cells [28] (DSMZ GmbH, Braunschweig, Germany; DSMZ no.: ACC 282) of approximately 13 µm diameter.

For the transport experiments, harvested cells are washed and aliquoted in buffer (autoMACS Running Buffer; Miltenyi Biotec B.V. & Co. KG; Bergisch Gladbach, Germany) to approximately 6 × 10^4^ cells/mL. Individual samples of 500 µL each are prepared in reaction vessels. One sample of each culture bottle is set aside and not transported for the negative control. Another sample is set aside as positive control and, before staining, is treated with Triton^TM^ X-100 (Merck KGaA, Darmstadt, Germany), which disrupts the cells’ membrane. Cells are transported with five different actuation settings as indicated in Table 1. Three individual samples are transported per setting and pump, leading to a total of 15 samples per setting. Results are averaged over all transported samples for each setting.

The experimental setup for the cell transport is depicted in Figure 2b. The pump’s inlet and outlet are connected with the inlet and outlet reservoir using flexible tubing with 4 cm length and 1.4 mm inner diameter. The inlet reservoir contains 500 µL of cell solution. To assure sufficient resuspension of cells, each reservoir is agitated carefully prior to transport that is completed in less than ten seconds. The cells are transported with the desired setting and collected in a reaction vessel. The solution is pumped one single time and not circulated or transported repeatedly. For cleaning, we passively flush the pumps with 20 mL buffer (autoMACS Running Buffer; Miltenyi Biotec B.V. & Co. KG; Bergisch Gladbach, Germany) between samples with different actuation signals. After flushing, 500 µL of rinsing solution are collected and analysed for the number of cells to assure that the following sample is not influenced by remaining cells in the pump.

#### Propidium Iodide Cell Viability by Flow Cytometry

To evaluate the amount of intact and dead cells, samples are stained with propidium iodide (PI) solution (Miltenyi Biotec B.V. & Co. KG; Bergisch Gladbach, Germany), a nucleus staining agent that is not able to penetrate an intact membrane of viable cells. To each sample, including the positive control and “Non-pumped” samples (negative control), 6 µL PI is added and incubated for 15 min. All samples are washed and distributed to a 96 well plate for automatic flow cytometry measurements with the MACS Quant Analyzer 10 Optical FlowCytometer (Miltenyi Biotec B.V. & Co. KG; Bergisch Gladbach, Germany). To account for time effects, the positive and negative control are analysed both before and after all other samples with no visible change.

The collected data are gated for single cells using the height versus forward scatter area. The samples for a given actuation signal are averaged over all five pumps. For comparison of two sample groups that include 15 individual samples with approximately 30,000 cells each, we use a two-tailed *t*-test with a *p*-value of 0.02.

## 3. Results and Discussion

Cell transport with very limited available space is a challenging task. Both damage on the cells caused by the pump as well as pump degradation due to cell transport are possible. With a detailed experimental consideration, we intend to clarify possible influences and provide the basis for further improvements.

### 3.1. Pump Characterisation

Micro diaphragm pump characterisation includes the evaluation of the general properties of the pump as well as an investigation of the single stroke with different actuation mechanisms. The later generates information on the fluidic efficiency of each actuation and allows us to adapt the actuation signal to a fluidically optimal transport with less influence on the cells.

#### 3.1.1. General Micro Diaphragm Pump Characterisation

To estimate the impact of cell transport on the diaphragm pump itself, all devices are characterized prior to and after the cell transport. It is possible for cells to accumulate in the pump chamber as well as in the dead volume around the valves and therefore lead to a pump degradation. A change in stroke or passive as well as active flow characteristics allows for better understanding of failure mechanisms.

**Figure 3:**
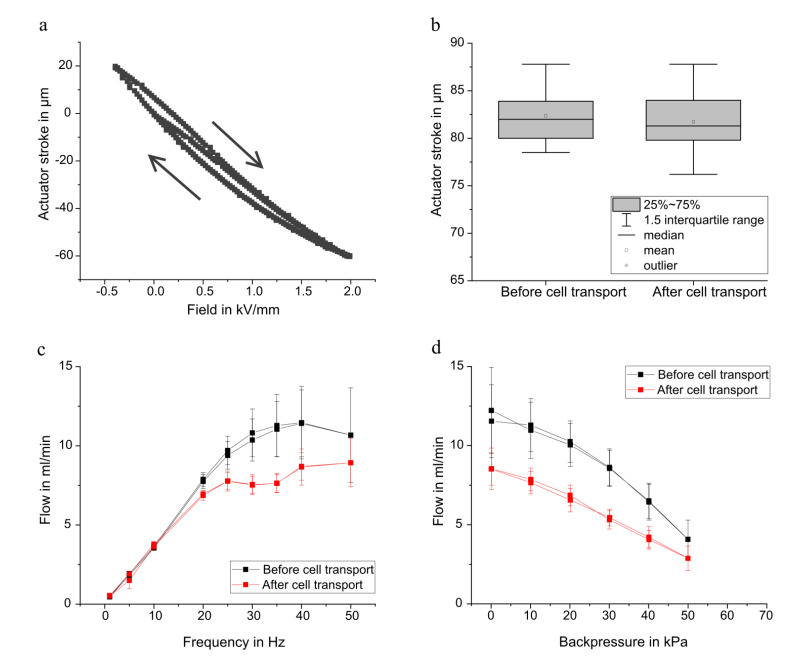
Mechanical and fluidical characterization of the tested micro diaphragm pumps. (**a**) The actuator displacement shows the typical piezoelectric hysteresis. Adapted from Bußmann et al. [25]. (**b**) There is no change in the total stroke height before and after the cell transport. (**c**) The frequency-dependant flow rate with 14 kPa backpressure at −80/300 V (corresponding to −0.4/1.5 kV/mm) sinusoidal actuation remains nearly unchanged in the linear regime, but changes for high frequencies. The maximal achievable flow rate after cell transport is smaller. (**d**) Backpressure dependant flow rate at −80/300 V sinusoidal actuation with 30 Hz.

The exact movement of the diaphragm is a crucial part of the pump’s functionality. A typical stroke measurement is shown in Figure 3a, with the clear piezoelectric hysteresis. The stroke height determines the stroke volume and therefore the achievable flow rate of the system. The stroke measurement allows to detect mechanical constraints to the actuator’s movement. The average total stroke after cell transport is 81.7 ± 3.9 µm and not significantly changed compared to the initial average stroke of 82.4 ± 3.4 µm (Figure 3b). These findings indicate that cells do not agglomerate in the chamber and block the actuator movement.

Figure 3 shows the frequency dependant flow rate of the used steel micropumps. The flow increases linearly with the frequency until dynamic damping effects in the chamber become dominant and the curve plateaus. In the non-linear regime, the flow strongly depends on the fluidic periphery [29]. Therefore, a performance comparison should take place in the linear regime. The flow prior to cell transport experiments matches the expectation based on former pump batches [25]. After cell transport, the average flow up to 10 Hz is equal to the flow before the exposure to cell solution. The ability to achieve the same flow at low frequencies indicates no significant change in the behaviour of the passive flap valves. Low frequencies with sinusoidal actuation lead to a slow actuator movement and therefore low pressure gradients. The valves are opened and closed comparatively slowly with low force. A bad valve quality is therefore often visible in the low frequency range. The assumption of constant valve performance before and after the cell transport is substantiated by the leakage rates, which only changed for one out of six pumps. The leakage rate at 50 kPa differential pressure of the pumps before cell transport is below 30 µL/min for all samples. After transport, it remains unchanged for all but one pump that shows a drastically increased leakage rate of 260 µL/min. This increase is potentially caused by fibres caught in the passive flap valves that were visible in the optical inspection and probably transported into the pump during use outside of the clean room environment. The leakage measurement always detects the leakage over both, the inlet as well as the outlet valve. If only one valve is sufficiently tight, the overall leakage is small. Significant backflow in active pump mode is still possible, if the other valve is leaking. 

For higher frequencies, the average flow decreases and the end of the linear region is lower. A volume change in the chamber, due to agglomeration of cells or particles, is a possible explanation for the detected change in the fluidic performance. Furthermore, increased leakage through the passive flap valves can change the flow behaviour. However, as described above, increased leakage has a large influence on the flow at small frequencies and would be visible in the linear range.

The decrease in flow for higher frequencies after cell transport compared to the initial performance is also visible in the backpressure measurements of the pumps. With 30 Hz, sinusoidal actuation, the flow at 0 kPa is approximately 3 mL/min lower after the cell transport. The extrapolated blocking pressure however remains unchanged with approximately 70 kPa before and after cell transport. This indicates the same stiffness of the actuator and therefore nor degradation of the ceramic itself or the adhesive connection during the experiments.

#### 3.1.2. Single Stroke Characterisation

The single stroke measurement with varying actuation signals allows to estimate the fluidic efficiency of the transport. A single actuator stroke requires approximately 2 mJ and single stroke power consumption of the pump itself (without driving electronics) is independent of the waveform and frequency [25]. From the energy required for one pump stroke and the volume transported per stroke, we can calculate the power consumption per dosed volume. Since the energy per stroke is constant, it depends on the single stroke volume that again depends on the actuation signal.

A perfectly rectangular actuation generates the fastest actuator movement and therefore the fastest pressure built-up. Consequently, the actuation of the passive flap valves is rapid and backflow through insufficiently closed valves is minimized [30]. A lower flank steepness, as is the case for sinusoidal actuation, leads to a slower pressure built-up and slow opening and closing of the passive valves. It therefore allows a portion of the transported fluid to move backwards through the valves [30]. A steeper flank therefore leads to a higher stroke volume. However, the fast actuator movement also increases the strain on both, the pump as well as the dosed medium, since it leads to high mechanical stress in the piezoelectric actuator and increases the fluidic shear stress.

**Figure 4:**
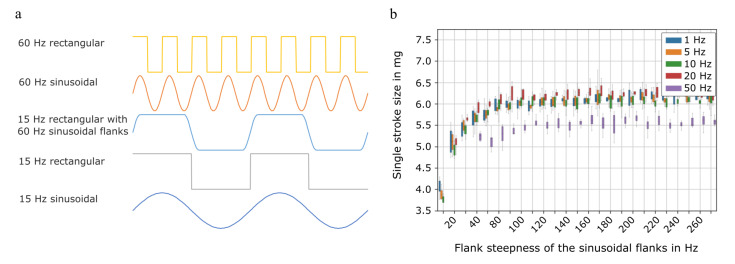
(**a**) Comparison of the different actuation signal chosen for cell transport. The hybrid signal of 15 Hz rectangular actuation with 60 Hz sinusoidal flanks is a mix of the depicted 15 Hz rectangular waveform and the 60 Hz sinusoidal waveform. (**b**) Single stroke volume transported by a micropump dependent on the actuation frequency and the steepness of the sinusoidal flanks.

A trade-off between fluidic efficiency and strain on pump and medium can be a hybrid actuation based on a rectangular waveform with sinusoidal flanks. Figure 4a shows the resulting waveform in comparison to other waveforms: the hybrid signal is a mix between a rectangular signal with a given frequency and a sinusoidal signal of a higher frequency. In order to evaluate which flank is appropriate, we determined the single stroke volume for several actuation frequencies with varying flanks. A frequency dependence of the single stroke volume is expected due to fluidic damping in the chamber at higher frequencies and flow rates. Figure 4b depicts the dependence of the single stroke volume on the actuation frequency and flank steepness. The single stroke volume is reduced for 50 Hz actuation frequency compared to slower actuation, whereas it is independent of the actuation frequency up to 20 Hz. 

**Figure A2:**
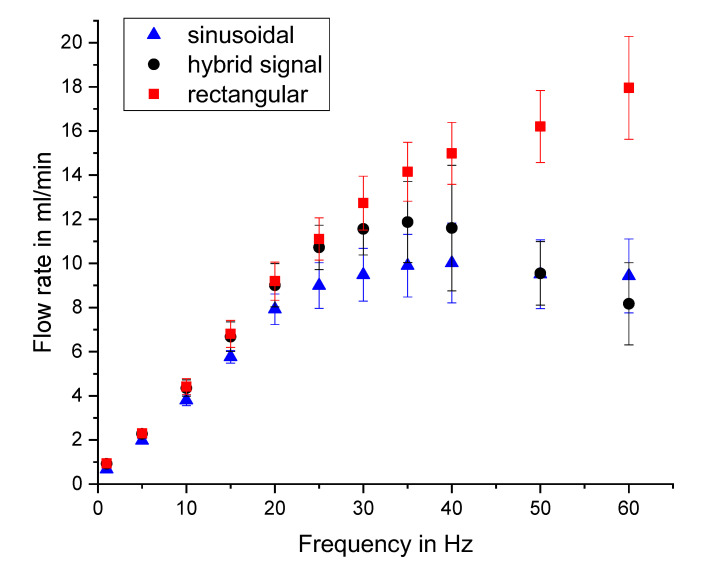
Comparison of the fluidic performance of the tested micropump type with different actuation signals. Frequency dependent flow rate at 14 kPa backpressure and an actuation amplitude of −89/300 V. The compared waveforms are a rectangular wave, sinusoidal wave, and a hybrid signal that consist of a rectangular waveform with 60 Hz sinusoidal flanks as has been used for cell transport. The error bars depict the standard deviation of five individual pumps.

For all tested frequencies, the single stroke volume plateaus for sufficiently steep flanks. The required flank steepness depends on the actuation frequency. While for actuation with 1 to 20 Hz, a flank steepness of approximately 60 Hz is sufficient, the single stroke volume at 50 Hz only plateaus at steeper flanks of approximately 90 Hz. The results are reproducible for all four evaluated pumps. These results indicate that the fluid transport is as efficient with sufficiently steep flanks as it is with rectangular actuation, however, the flank steepness needs to be comparatively high for higher actuation frequencies. The resulting flow rate for specific waveforms is verified with the measurement of the frequency dependant flow rate. Up to 25 Hz actuation frequency, the hybrid signal achieves the same flow rate as the rectangular signal. The experimental results are presented in Figure A2 in the Appendix B. Based on these findings, a 15 Hz hybrid signal with 60 Hz sinusoidal flank is chosen as one of the signals used for cell transport. This allows to compare the impact when the pump is actuated with sinusoidal, rectangular, or the hybrid signal.

### 3.2. Cell Transport

Viable cells are sensitive to their environment, and transport with a micro diaphragm pump can damage them in different ways. A first important indicator to assess the feasibility of cell transport with micro diaphragm pumps is the recovery rate of cells after transport. Neither the total cell concentration nor the percentages of cells within the gate deviate significantly between the negative control sample and the transported samples. Cells therefore do not accumulate within the pump, which corresponds well to the results of the electromechanical and fluidic characterisation of the pumps presented above.

#### 3.2.1. Consideration of Effects Influencing the Cell Viability

Pumping can have a mechanical impact on the transported cells due to moving parts in the flow path. For instance, cells can get caught in between the valve and its seat. However, this scenario is unlikely: one pump stroke, and therefore one opening and closing motion of the valves, transports approximately 10 µL of fluid. The total fluid volume between the valve and its seat when the valve starts closing is calculated based on the valve geometry: the surface of the valve seat of 0.23 mm^2^ and a valve opening of maximal 50 µm. It is approximately 0.01 µL for each valve. Therefore, if we assume that the cells are uniformly distributed and that all cells that are between the valve and its seat when the valve starts closing get caught in the valve, this would only affect 0.2% of the transported cells. We thus assume that the damage caused by hard-hard touch squeezing is neglectable.

It is also imaginable that cells become damaged when they come into contact with sharp edges. In particular, the etched valve structure or the drilled inlet and outlet access constitute such sharp edges. However, fluid flow around the cells causes fluidic drag thus their fluidic resistance is high and the cells move towards the middle of the flow path. Hence, the cells likely flow around the edges without contact. The effect is already shown by Ozbey et al. [31] who observe cells moving towards the centre of the channel in laminar flow.

A likely influence on the cell viability is the experienced fluidic shear stress. Studies discuss the impact of shear on non-adherent cells and indicate that the size as well as the deformability of a cell have an influence on its behaviour when subjected to shear stress [31,32,33].

To generate a first understanding of the shear force the cells are subjected to when passing through the pump, we consider the analytic description of a strongly simplified model. We aim to compare the influence of specific geometries in the pump, e.g., chamber and valves, as well as different actuation signals. We do not intend to give a quantitative analysis of shear forces.

The valve as well as the pump chamber have a high aspect ratio: The valve seat is 300 µm long and the valve opens 50 µm; the pump chamber is approximately 100 µm to 150 µm high (in the upwards position of the actuator) and we consider the distance of 5000 µm between the two valve openings as the gap length. We assume laminar gap flow in these geometries. Furthermore, for simplicity, we only consider the liquid medium itself and do not specifically calculate the velocity of the cells in this flow. This strong simplification allows a first comparison of geometries and actuation signals.

The shear stress of the laminar flow is given by

(1) $$\sigma =\eta \frac{\partial v_{x}}{\partial z}$$

with the viscosity of the liquid *η* and the flow velocity *v_x_*. Furthermore, the velocity profile of the laminar gap flow is parabolic

(2) $$v_{x}\left(z\right)=\frac{\Delta p}{2\eta L_{gap}}\left(h_{gap}z-z^{2}\right)$$

and depends on the pressure difference ∆p, the gap length *L_gap_*, the gap height *h_gap_*, and the viscosity of the liquid *η*. When the pump is active, the movement of the actuator diaphragm generates the pressure difference ∆p that drives the fluid through the gap and therewith generates the fluidic shear forces. The maximal pressure that the bending actuator can achieve is its blocking pressure. This pressure is generated when the actuator is subjected to a rectangular actuation and the movement is extremely fast. Since the maximal pressure occurs during this fast actuator displacement, the pressure peak is independent of the actuation frequency for rectangular actuation. The maximal pressure difference depends on the bending actuator’s geometry and used material and can be calculated with the analytical model presented by Herz et al. [34].

For sinusoidal actuation, the voltage change and therewith the actuator movement is considerably slower than for rectangular actuation. The time constant of the actuator movement and the fluid movement through the passive valves therefore converge and the maximal pressure peak decreases. Since the speed of actuator movement depends on the actuation frequency for sinusoidal actuation, the generated pressure difference is also a function of the actuation frequency. Similarly, for the hybrid signal, the maximal pressure depends on the speed of the actuator’s movement and therefore on the sinusoidal flank steepness of the signal. Based on the model by Herz et al. [34] the analytic description of the pressure peak of the sinusoidal actuation can be derived. However, the equation cannot be solved analytically and a numerical description will be part of future work. However, the analytical model allows for a qualitative comparison that shows that the shear force is maximal for rectangular actuation and minimal for sinusoidal actuation with a small frequency. Due to the relation between the maximal pressure and the flank steepness, the pressure difference and therewith the shear forces in this model are equal for sinusoidal actuation with 60 Hz and the hybrid signal with 60 Hz sinusoidal flanks.

To identify the most relevant pump geometries, the shear stress in the valve and the chamber are compared for rectangular actuation. For the simplified model, we assume gap flow in the valve as well as in the pump chamber as described above. Furthermore, we assume that the maximal pressure difference applies to both the chamber and the valve, even if in reality the pressure is split.

(3) $$\sigma =\frac{\Delta p}{2L_{gap}}\left(h_{gap}-2z\right)$$

(4) $$\sigma_{max}={\left.\sigma \right|}_{z=0}=\frac{h_{gap}}{2L_{gap}}\Delta p$$

Based on Equation (4) and the geometry of the flow path (the valve seat length *L_valve_* = 300 µm and valve opening *h_valve_* = 50 µm, as well as pump chamber length *L_chamber_* = 5000 µm and pump chamber height *h_chamber_* = 100 to 150 µm), the ratio of shear in the chamber and the valve can be estimated with

(5) $$\frac{\sigma_{chamber}}{\sigma_{valve}}=\frac{L_{valve}}{L_{chamber}}\frac{h_{chamber}}{h_{valve}}$$

and is approximately 0.12 to 0.18. As can be seen from Formula (5), the ratio is independent of the pressure difference. The estimation shows that the shear stress at the valve unit is larger compared to the shear stress in the pump chamber. To further reduce the impact of the pump on cells, it is thus necessary to adapt the valve geometry to reduce the occurring shear stress in this area.

#### 3.2.2. Viability of K-562 and Jurkat Cells after Transport

In this preliminary study, we only detect the immediate influence of the pump on the cells, since we measure the percentage of intact cells compared to the negative control. The influence of the transport on the long-term viability of the cells are not yet examined and will be part of future studies.

**Figure 5:**
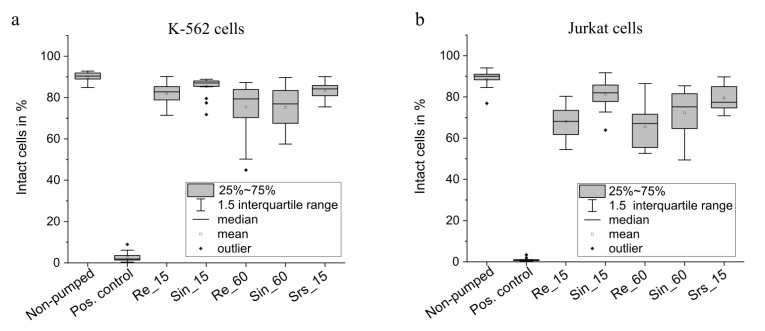
Percentage of viable cells after gating for single cells in the control samples as well as in the transported cell solution for K-562 cells (**a**) and Jurkat cells (**b**). Data for each actuation type include 15 individual samples.

Both cell types are impacted by the pumping process. For K-562 cells, the non-pumped (negative) control samples contains 89.9 ± 2.6% intact cells; for Jurkat cells it contains 88.6 ± 5.2% intact cells. Figure 5 shows the change of the percentage of intact cells due to transport with the micro diaphragm pump with different actuation signals. The mean percentage of intact cells is slightly lower for all transported samples compared to the non-pumped control and the decrease is significant with *p* ≤ 0.02.

The various actuation signals cause different damage of the K-562 cells. The largest decrease of intact cells is caused by the actuation with 60 Hz and the use of sinusoidal or rectangular actuation does not have a significance influence on the impact. With an average of 75.4% intact cells, there are 14.5 percentage points less intact cells compared to the non-pumped control. A significantly lower damage (*p* ≤ 0.02) is caused by sinusoidal actuation with 15 Hz, which is the smoothest actuation. The mean percentage of intact cells is 85.4 ± 4.6% and therefore only 4.5 percentage points lower than the non-pumped control. The smaller impact can be due to the overall lower flow rate and thus reduced shear force. Furthermore, the maximal speed of the diaphragm and therewith the maximal pressure difference is decreased and evokes a smaller maximal pressure gradient. The average damage caused by a rectangular actuation with 15 Hz shows, that the faster actuator movement causes more damage. However, the difference is only significant with *p* ≤ 0.05. As described above, for rectangular actuation, the maximal pressure difference does not depend on the actuation frequency. Therefore, the analytical model cannot explain the different impact of 60 Hz and 15 Hz rectangular actuation on the transported cells. However, the average overall flow rate and thus average shear in the pump and periphery are larger for 60 Hz actuation. The transport with the hybrid signal (15 Hz rectangular signal with 60 Hz sinusoidal flanks: srs_15) imposes a slower actuator movement compared to 15 Hz rectangular actuation while maintaining the fluidic performance. The impact on the cells is also slightly reduced, but the difference is not significant.

The impact of the pump on Jurkat cells is more pronounced. The diameter of the two cell lines is similar, though the deformability is different. Since the deformability determines the path of the cell in the fluidic channel [32], the increased impact on Jurkat cells can among others be caused by a difference in the experienced fluidic shear stress. The largest decrease of intact cells occurs after transport with a rectangular actuation of 60 Hz, which is the most stressful actuation. It generates the fastest actuator movement, highest pressure peak and the highest overall flow. On average, 65.6 ± 10.3% of cells stay intact after this transport, nearly one quarter of cells less than the control. A similar large impact (no significant difference, *p* ≥ 0.05) is caused by rectangular actuation with only 15 Hz (68.0 ± 8.0%). As described above, the maximal pressure peak and therefore the maximal fluidic shear stress caused by the pressure peak that pushes fluid through the valve channel, do not depend on the applied frequency for rectangular actuation. The similar reduction of viable cells for 15 Hz and 60 Hz rectangular actuation indicates that this maximal shear stress has a significant influence on the transported cells. For both actuation frequencies, sinusoidal actuation causes less damage with 81.3 ± 7.6% intact cells for 15 Hz actuation and 72.3 ± 10.3% for 60 Hz. However, the sinusoidal actuation with a frequency of 60 Hz still stresses the cells and the impact is not significantly different to rectangular actuation (*p* ≥ 0.05). The influence of the transport with hybrid actuation (15 Hz rectangular actuation with 60 Hz sinusoidal flanks) on the cells is very similar to the 15 Hz sinusoidal actuation and the difference is not significant with *p* ≥ 0.05. According to the analytical model described above, the generated pressure peak and imposed shear are the same for the hybrid actuation and 60 Hz sinusoidal actuation, since the flank steepness of the two signals is equal. The different impact of the hybrid actuation and 60 Hz sinusoidal actuation indicate that not only the maximal experienced shear, but also the average overall flow rate impact the cells. The average flow is higher for the 60 Hz sinusoidal actuation than for the hybrid signal (Figure A2, Appendix B).

Overall, the transport with 15 Hz hybrid signal has a similar impact on the cells as the transport with sinusoidal actuation (Figure 5) while maintaining the fluidic performance of rectangular actuation as shown by the comparison of the single stroke volume as well as the flow characterisation presented in Figure A2, Appendix B. The hybrid actuation therefore offers the possibility of efficient cell transport while minimizing the damage. However, for both cell types, the impact is still significant with approximately 7.3 and 4.6 percentage points less intact cells compared to the control sample for Jurkat and K-562 cells respectively. It is thus necessary to further optimize the pump systems with respect to the exact valve geometry, the pump chamber, as well as the inlet and outlet channel.

## 4. Conclusions

Overall, the standard characterisation of the pumps before and after cell transport shows a small degradation but no complete loss of functionality. It is important to notice that during the transportation experiments the pumps were not used in a cleanroom environment and therefore not only exposed to cell solution but also to contamination such as fibres. The optical examination of the valves did show fibres in several pumps. Hence, it is not possible to distinguish degradation caused by cells from other influences and further evaluations are necessary to assess possible pump degradation. In preliminary experiments we plan to evaluate the particle resistance of the micro diaphragm pumps with the transport of polystyrene particles, before we evaluate the influence of cells.

Furthermore, a more detailed investigation of the pump’s impact on the cells is necessary. In this preliminary experiment, we only evaluated immediate damage to the cells by staining. However, a long-term effect, e.g., reduced activity, is very likely. We will therefore conduct further evaluation including cell activity assays and long-term evaluation.

Overall, the results on pump performance as well as the impact on the cells are very promising. The impact on the cells can be further minimized with the optimization of the pump’s geometry. The analytical estimation of shear forces in the pump shows higher shear forces in the valve gap compared to the chamber. To reduce this shear stress, an optimisation towards larger valve opening can be envisaged by a reduction of the stiffness of the flap valve. Furthermore, shear stress can be reduced by adapting the channel size of the inlet and outlet as well as the pump chamber geometry in terms of structure or height. With those measures we expect to further limit the damage caused by the pump and enable the development of improved and miniaturized transport systems.

## Figures and Tables

**Table 1:** Used actuation signals for the transport of cells. The waveforms are described in further detail in Section 3.1.2.

	Waveform	Frequency in Hz	Abbreviation
Setting 1	Rectangular	15	Re_15
Setting 2	Sinusoidal	15	Sin_15
Setting 3	Rectangular with 60 Hz sinusoidal flanks (hybrid actuation)	15	Srs_15
Setting 4	Rectangular	60	Re_60
Setting 5	Sinusoidal	60	Sin_60

**Waveform**

**Frequency in Hz**

**Abbreviation**

**Figure A1:**
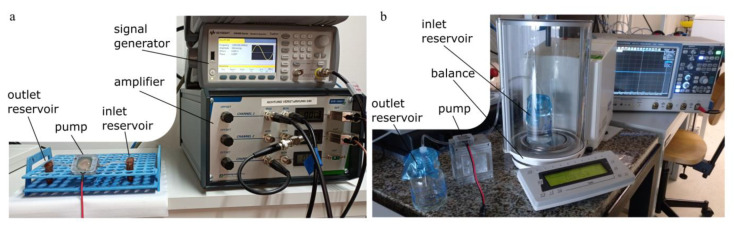
(**a**) Experimental setup of the cell transport including an inlet and outlet reservoir as well as the electric actuation of the pump. (**b**) Preliminary gravimetric setup to detect the single stroke volume of the piezoelectric micropump. The setup was slightly improved for the final experiments.

## Data Availability

The actuator stroke, fluidic test, and simulation data that support the findings of this study are available in Fordatis–Research Data Repository of Fraunhofer-Gesellschaft with the identifier http://dx.doi.org/10.24406/fordatis/160 (accessed on 26 November 2021).

## Appendix A

### Experimental Setup for Cell Transport and Gravimetric Measurement

The setup for cell transport experiments (Figure A1a) is simple with the pump being connected with flexible tubing with the inlet and outlet reservoir. The gravimetric setup was evaluated in a preliminary setup as shown in Figure A1b. In this setup, the pump transports liquid from the inlet reservoir on the balance towards an outlet next to it. The actuation signal of the pump is verified with an oscilloscope. This setup was improved for the experiments in this work as shown in Figure 2.

## Appendix B

### Flow Characterisation with Different Actuation Waveforms

The mean frequency dependant flow rate of five pumps shows an influence of the actuation waveform (Figure A2). The rectangular actuation and the hybrid actuation (rectangular with 60 Hz sinusoidal flanks) show a similar flowrate up to 25 Hz actuation frequency. For higher frequencies, the rectangular signal achieves clearly higher flow. However, a rectangular actuation can damage the actuator ceramic especially during long term use with liquid media, which is why the hybrid signal can be a good tradeoff. The increase of flow rate compared to sinusoidal actuation is 16% for 15 Hz, which is the frequency used for cell transport.

The periphery has an influence on the flowrate, especially for higher actuation frequencies. Since the flow characterisation takes place in a large setup with comparatively long tubes and sensors that depict a flow resistance, the real flow rate during cell transport (with short tubes and no additional elements) is potentially even higher for the transport with 60 Hz.
